# An Acridine-Based Fluorescent Sensor for Monitoring ClO^−^ in Water Samples and Zebrafish

**DOI:** 10.3390/s20174764

**Published:** 2020-08-23

**Authors:** Su Chan Lee, Soyoung Park, Haeri So, Gyudong Lee, Ki-Tae Kim, Cheal Kim

**Affiliations:** 1 Department of Fine Chemistry, Seoul National University of Science and Technology, Seoul 136-741, Korea; rlthro456@naver.com (S.C.L.); pp113833@hanmail.net (S.P.); gofl0988@naver.com (H.S.); 2 Department of Environmental Engineering, Seoul National University of Science and Technology, Seoul 136-741, Korea; rbehd8024@gmail.com

**Keywords:** acridine, fluorescent chemosensor, hypochlorite, zebrafish, theoretical calculations

## Abstract

A novel acridine-based fluorescent chemosensor, **BK** ((E)-2-((acridine-9-ylimino)methyl)-N-benzhydrylhydrazine-1-carbothioamide), for monitoring ClO^−^ was prepared. The sensor **BK** was synthesized by introducing a new synthetic route of making aldehyde group using formic hydrazide. Probe **BK** displayed notable fluorescence quenching in the presence of ClO^−^ and showed a great selectivity over other guest analytes. The detection limit was calculated to be 7.65 μM. Additionally, **BK** was satisfactorily applied for sensing ClO^−^ in water samples and zebrafish.

## 1. Introduction

Interest in quantification of reactive oxygen species (ROS) has grown owing to the indispensable role played by ROS in pathological and physiological processes [1,2,3,4,5]. For instance, recent research revealed that cancer cells steadily produce high concentrations of intracellular ROS, owing to carcinogenic deformation [6,7,8]. Hypochlorite (ClO^−^) is one of the ROS, which was produced from the oxidation reaction of Cl^−^ and H_2_O_2_ catalyzed by the heme protein myeloperoxidase [9,10,11]. Hypochlorite is well known as a bactericidal agent because of the capability to kill the deleterious bacteria and pathogens [12,13,14,15]. However, the abnormal levels of ClO^−^ in life systems are related to various diseases like cystic fibrosis, neuron degeneration, kidney disease, arthritis, atherosclerosis, and cancer [16,17,18,19,20]. Hence, there is urgent need to develop effective and dependable sensors for detecting ClO^−^ to understand the role of hypochlorite in organisms.

Until now, various analytical methods for the sensing of ClO^−^ have been established, such as electrochemistry, colorimetry assays, spectrophotometry and fluorescent chemosensors [21,22,23,24]. Among the methods for detecting ClO^−^, fluorescence imaging techniques have various virtues like specificity, superior sensitivity, manageability and fast response times [25,26,27,28]. Hitherto, several probes having fluorophores have been developed for sensing ClO^−^ like 1,8-diaminonaphthalene, phenanthrene, BODIPY, anthracene, rhodamine, 1,8-naphthalimide, coumarin and fluorescein [29,30,31,32,33,34,35,36]. However, some of them have disadvantages like complicated synthetic routes, poor water solubility and being unsuitable for biological application. Thus, there is still a need to further exploit new fluorescent chemosensors for sensing ClO^−^ in vitro as well as in biological systems [37,38,39].

Acridine and its derivatives have been interesting subjects to researchers for a long time because of their ability to bind DNA and act as a good fluorophore [40,41,42,43,44]. In addition, benzhydryl isothiocyanate is water-soluble and used for a linker [45]. Therefore, we linked acridine moiety with benzhydryl isothiocyanate to develop a sensor having the unique photophysical, water-soluble and biocompatible properties for the detection of ClO^−^.

Herein, we represent a novel acridine-based fluorescent probe, **BK**, for detecting ClO^−^. The reaction of **BK** with ClO^−^ showed a fluorescent quenching in aqueous media. The sensing ability of the probe was investigated by fluorescent and UV-visible titrations. The sensing mechanism of **BK** towards ClO^−^ was also demonstrated via ESI-mass spectrometry and theoretical calculations. In addition, the **BK** probe was satisfactorily examined to monitor ClO^−^ in environmental water samples and zebrafish.

## 2. Experiments

### 2.1. Materials and Equipment

All reagents and solvents were purchased from Sigma-Aldrich. ^13^C NMR (100 MHz) and ^1^H NMR (400 MHz) data were provided on a Varian spectrometer. UV-vis and fluorescence measurements were performed on Perkin Elmer UV/Vis and fluorescence spectrometers. ESI-mass data were obtained by a single-quadrupole liquid chromatography detector (ACQUITY QDa).

### 2.2. Synthesis of KT (N-benzhydryl-2-formylhydrazine-1-carbothioamide)

An amount of 2 mmol of formic hydrazide and 2 mmol of benzhydryl isothiocyanate were dissolved in 5 mL of EtOH. The mixture was stirred until a white-colored powder was gained. The resultant powder was filtered and washed with ether. ^1^H NMR (deuterated DMSO, 400 MHz) δ (ppm): 11.4 (s, 2H), 10.7 (m, 2H), 8.35 (m, 4H), 8.25 (t, 2H), 8.15 (s, 2H), 8.05 (d, 2H), 7.95 (t,1H).

### 2.3. Synthesis of Sensor BK ((E)-N’’-(acridine-9-yl)-N’-((benzhydrylamino)(oxo-l4-sulfanylidene)methyl)formimidohydrazide)

An amount of 1 mmol of **KT** and 1 mmol of 9-aminoacridine (**ACR**) were dissolved in 5 mL ethanol. After the solution was stirred at 23 °C overnight, the yellowish powder was filtered and washed with methanol and ether. ^1^H NMR in DMSO-*d_6_*, δ: 8.51 (s, 1H), 8.43 (s, 1H), 8.39 (s, 1H), 7.8 (d, 2H, *J* = 8.8 Hz), 7.65 (t, 2H), 7.5 (m, 10H), 7.3 (m, 5H), 7.2 (s, 1H). ^13^C NMR: δ = 142.90, 140.87, 138.05, 132.82, 128.90, 128.26, 128.21, 128.02, 127.73, 124.05, 123.51, 122.71, 112.19, 61.93, 61.05 ppm. ESI-Mass: m/z calcd for [C_28_H_23_N_5_S – H^+^ + 2 Na^+^ + 2 Cl^−^]^−^: 576.08 found, 575.92.

### 2.4. General Procedure for the Spectroscopic Studies 

Probe **BK** stock solution (1 mM) was prepared in DMF. ClO^−^ stock solution was prepared by diluting NaClO (500 μmol, 12%, dissolved in H_2_O) in distilled water to make 100 mM. All anion and ROS stock solutions (1.0 × 10^−1^ M) were prepared in bis-tris buffer. The fluorescence and UV-vis data were measured in near 100% aqueous solution (bis-tris, 1 × 10^−2^ M, pH 7.0). 

### 2.5. Calculation of Quantum Yield

Quantum yield (Φ) was calculated by using quinine as a standard fluorophore (Φ_F:_ 0.54 in 0.1 M H_2_SO_4_). Equation for quantum yield is:

ΦF(X) = ΦF(S) (*AS FX /AX FS*) (*nX / nS*)^2^

(Φ_F(X)_: fluorescent quantum yield, x: unknown, *s*: standard, *A*: absorbance, *n*: refractive index of the solvent and *F*: the area of fluorescence emission curve).

### 2.6. Imaging Experiments in Zebrafish

Zebrafish embryos were cultured under the former conditions [46]. The 6-day-old embryos were incubated in E2 media replenished with 2 × 10^−5^ M of **BK** for 15 min and rinsed with E2 media to get rid of the remnant **BK**. One was a control group and the other group further treated with 50 μM of ClO^−^ for 15 min was prepared and washed with E2 media. Zebrafish were anesthetized by adding ethyl-3-aminobenzoate. The image of zebrafish was achieved by a fluorescence microscope.

### 2.7. Cytotoxicity in Zebrafish

Zebrafish larvae at 6-day old were exposed to 0 and 20 μM of **BK** and 20 μM of 9-aminoacridine in E2 media at 0.05% of DMSO for 20 min. Later they were diverted into 10 μg/mL of AO reagent (Sigma-Aldrich, St. Louis, MO, USA) in E2 media for 60 min. After the larvae were washed with E2 three times, the prepared larvae were mounted and photographed under a Leica fluorescence microscope (MZ10F, Singapore). Apoptosis was identified as an obvious bright spot.

### 2.8. Theoretical Calculations

DFT/TDDFT calculations on the basis of the hybrid exchange-correlation functional B3LYP were accomplished with the Gaussian 09 W program [47,48]. The 6-31G basis set was applied for all elements (C, S, N, O and H) [49,50]. Frequency calculations of **BK** and **ACR** (9-aminoacridine) were achieved to prove that the optimized forms displayed local minima, and imaginary frequencies were not observed at all. Cossi and Barone’s CPCM was employed to consider solvent effect of water [51,52]. To study the electronic properties of singlet excited states, TD-DFT was conducted for the ground state forms of **BK** and **ACR**. The twenty singlet-singlet excitations were quantitatively analyzed. GaussSum 2.2 was used to analyze the contributions of MOs [53].

## 3. Results and Discussion

**Scheme 1:**
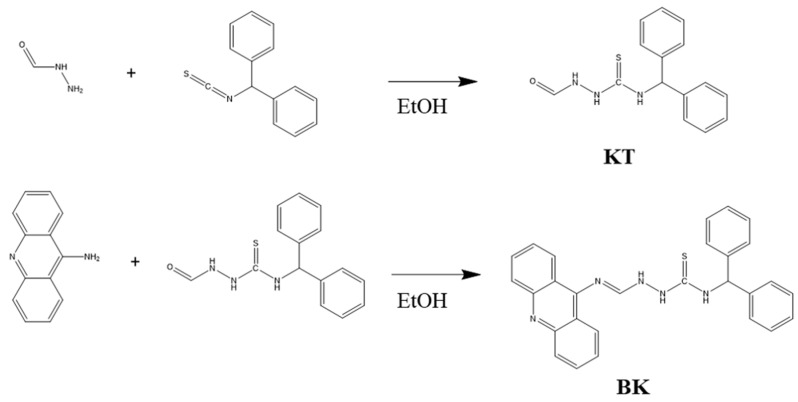
Synthesis of **BK** ((E)-2-((acridine-9-ylimino)methyl)-N-benzhydrylhydrazine-1-carbothioamide).

The synthetic route for chemosensor **BK** is outlined in Scheme 1 and it was successfully characterized by ^1^H and ^13^C NMR and ESI-MS. Sensing behavior of **BK** toward ClO^−^ was investigated by using several analytical tools like UV-visible and fluorescent spectroscopy, ^1^H NMR titration, and calculations.

### 3.1. Spectroscopic Investigations of BK to ClO^−^

**Figure 1:**
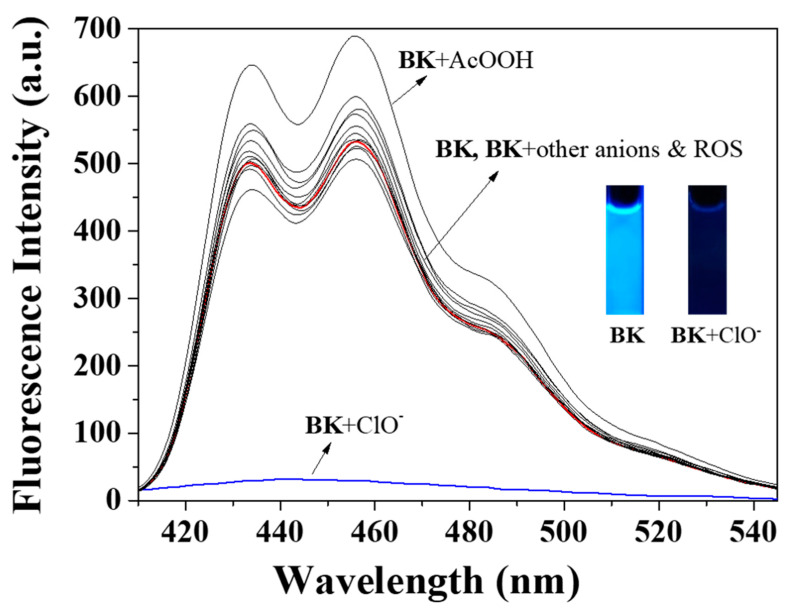
Fluorescence spectra of **BK** (1 × 10^−5^ M) with varied analytes (220 equiv). ROS: Reactive oxygen species.

Selectivity was one of the essential indicators for measuring capacity of fluorescent probe. Reactivity of **BK** to diverse analytes (N_3_^−^, Cl^−^, H_2_O_2_, CN^−^, S^2−^, I^−^, tBuOOH, SCN^−^, OAc^−^, Br^−^, AcOOH, H_2_PO_4_^−^, BzO^−^, F^−^, NO_2_^−^ and ClO^−^) in bis-tris buffer (1 × 10^−2^ M, pH 7.0, Figure 1) was tested to evaluate the selectivity for ClO^−^. With excitation at 350 nm, free sensor **BK** displayed a markedly strong fluorescence emission at 455 nm (Φ = 0.6659). Upon addition of each analyte into **BK** solution, only ClO^−^ induced the prominent fluorescent decrease (Φ = 0.0047) whereas other analytes did not show any noticeable changes. Therefore, the sensor **BK** could serve as a preeminent fluorescent sensor for ClO^−^.

**Figure 2:**
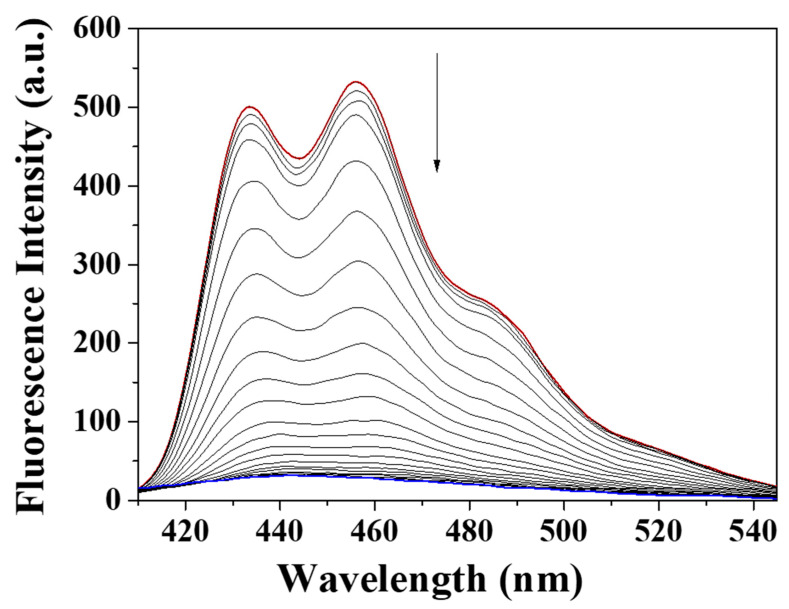
Fluorescent changes of **BK** (1 × 10^−5^ M) with different concentrations of ClO^−^.

**Figure 3:**
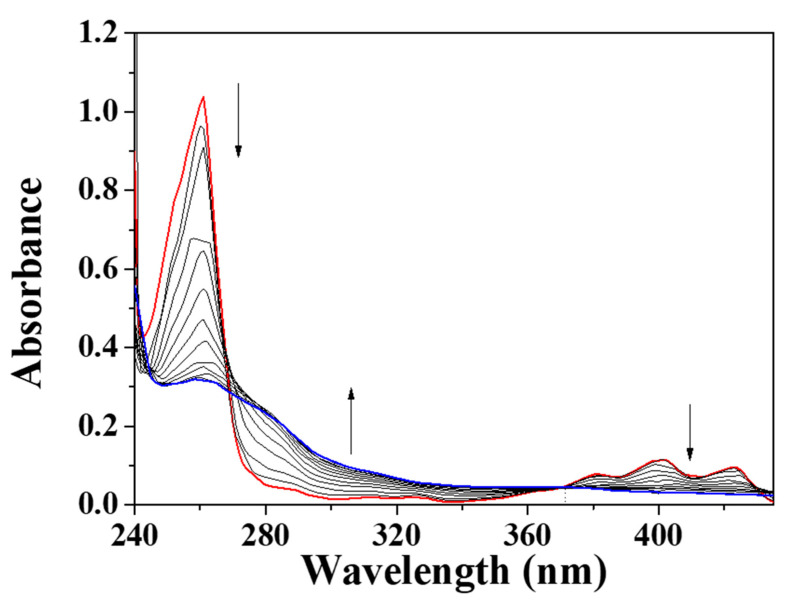
UV-vis variations of probe **BK** (1 × 10^−5^ M) with different concentrations of ClO^−^.

To examine the response of **BK** to ClO^−^, the spectral titrations were carried out (Figure 2). On the addition of different ClO^−^ amounts to **BK**, the fluorescence emission of 455 nm constantly decreased and was saturated at 220 equiv. The measured limit of detection (C_DL_ = 3σ/k) for ClO^-^ was 7.65 μM (Figure S1 in the Supplementary Materials). The UV-visible titration displayed that the absorbance of 280 nm constantly increased and the bands at 260 nm and 400 nm reduced with an obvious isosbestic point at 370 nm (Figure 3). 

**Figure 4:**
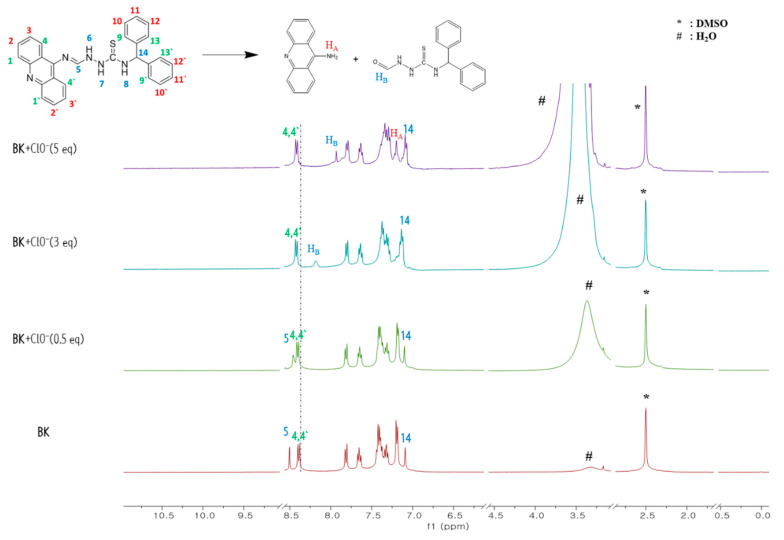
^1^H NMR titration of **BK** with ClO^−^.

To elucidate the detecting mechanism of ClO^−^, we conducted the ESI-mass experiment (Figure S2). The peak of 322.08 (m/z) was indicative of [**ACRO** + Cl^−^ + MeCN + 2H_2_O]^−^ [calcd, 322.10], indicating the cleavage reaction product of **BK** by ClO^−^. ^1^H NMR titration was carried out to observe the formation of **ACR** (Figure 4). With gradual addition of ClO^−^ to **BK**, the imine proton H_5_ disappeared, and the amine proton H_A_ of **ACR** and the aldehyde proton H_B_ of **KT** appeared. 

**Scheme 2:**
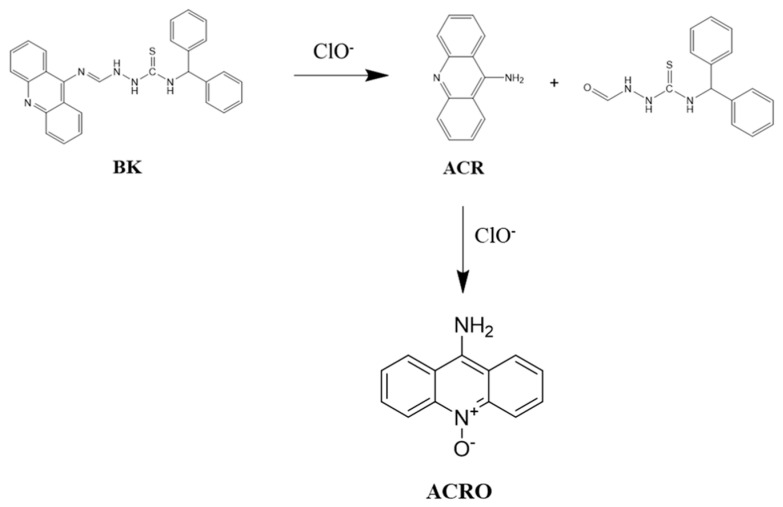
Cleavage sensing mechanism of **BK** by ClO^−^.

On the other hand, as 9-aminoacridine was highly fluorescent in nature, we examined the interaction of 9-aminoacridine with ClO^−^. As shown in Figure S3, ClO^−^ quenched the fluorescence of 9-aminoacridine, most likely due to an oxidation reaction. These observations led us to propose that the probe **BK** was cleaved by ClO^−^ via the cleavage process of the C=N bond to produce 9-aminoacridine. Then, the fluorescent 9-aminoacridine was subsequently quenched by ClO^−^ (Scheme 2). 

**Figure 5:**
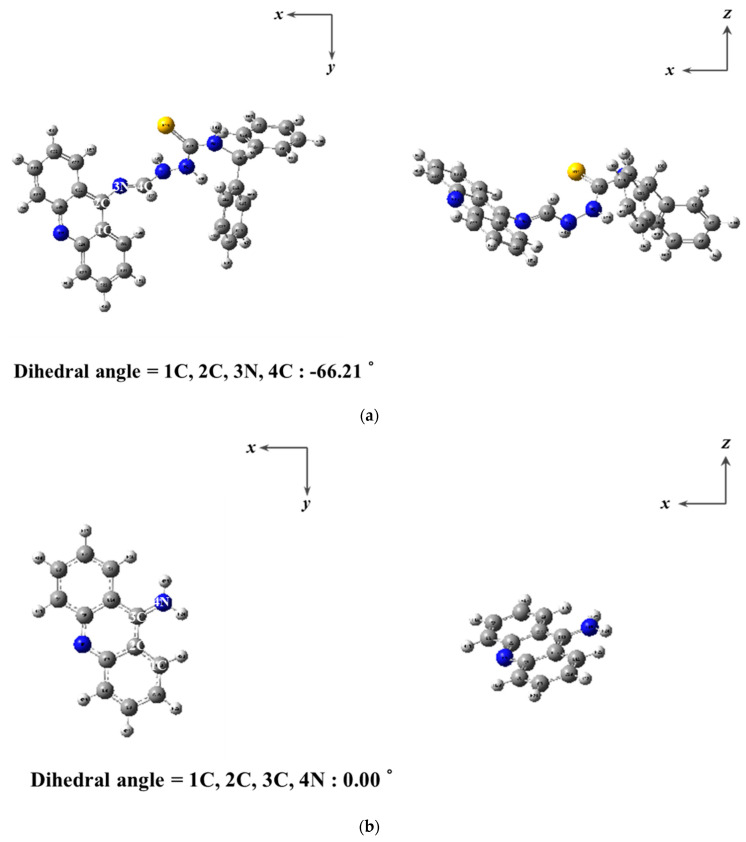
Energy-minimized forms of (**a**) **BK** and (**b**) 9-aminoacridine (**ACR**).

To further comprehend the cleavage sensing mechanism of **BK** by ClO^−^, computational calculations were achieved. Energy-optimized forms of **BK** and **ACR** were analyzed with DFT/B3LYP/6-31G (d,p) basis sets (Figure 5). Using the optimized forms of **BK** and **ACR**, TD-DFT calculations were performed for analyzing transition energies and molecular orbitals (Figures S4–S6). The MOs of **BK** at the first lowest excited state were identified as the HOMO → LUMO transitions (391.73 nm, Figure S5), which turned out to be π → π* transitions. For **ACR**, the MOs at the first lowest excited state were identified as the HOMO → LUMO (391.84 nm, Figure S6), which turned out to be π → π* transitions. In addition, the decreased oscillator strength of **ACR** was corresponded with the decreased UV-vis absorbance. These outcomes drove us to elucidate that fluorescent sensor **BK** was quenched due to the cleavage of the C=N bond.

**Figure 6:**
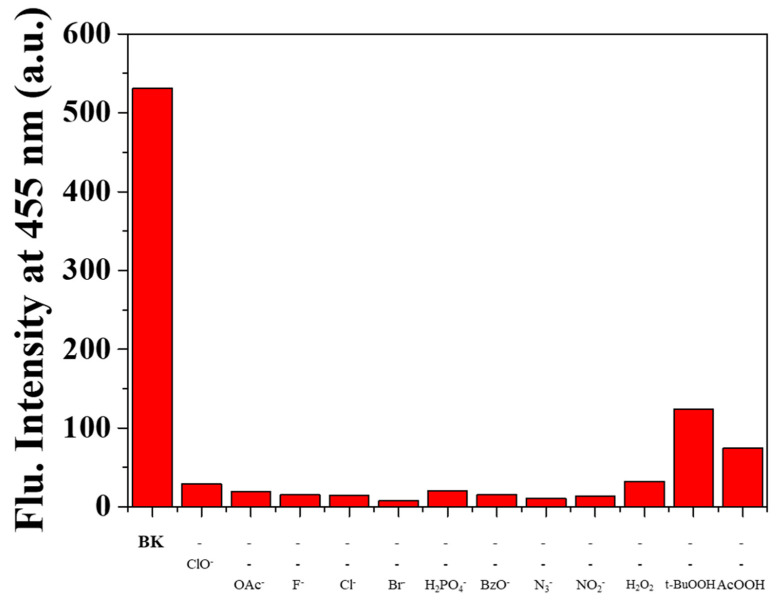
Competitive selectivity of **BK** (1 × 10^−5^ M) to ClO^−^ (220 equiv) with other analytes (220 equiv).

Another vital indicator in the sensing process is the ability of the fluorescent probe not to be disturbed by the competitive analytes. When **BK** was exposed with both ClO^−^ and diverse analytes (N_3_^−^, Cl^−^, H_2_O_2_, tBuOOH, OAc^−^, Br^−^, AcOOH, H_2_PO_4_^−^, BzO^−^, F^−^ and NO_2_^−^), the guest analytes did not hinder the sensing of ClO^−^ (Figure 6). Only, the presence of tBuOOH and OAc^−^ showed slight interference in determining ClO^−^. CN^−^, S^2^^−^, I^−^ and SCN^−^ were excluded from the competition because they reacted directly with ClO^−^.

**Figure 7:**
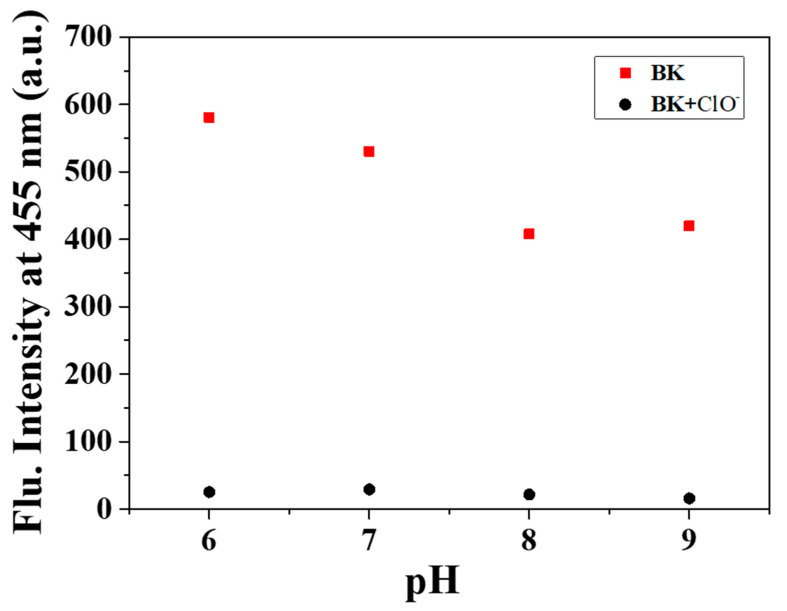
Fluorescence intensity (at 455 nm) of **BK** with ClO^−^ at pH values from six to nine.

The pH was a vital condition related with physiological processes and even cellular behaviors. Thus, we checked the effect of pH on sensing property of **BK** for ClO^−^ at pH ranging from six to nine (Figure 7). **BK** displayed significant fluorescence intensity at the pH range of six to nine. The intensity of **BK** treated with ClO^−^ was completely quenched at pH six to nine. These observations indicated that **BK** could be applicable as a probe for detecting ClO^−^ at pH six to nine.

To explore practical utility of **BK**, the application of **BK** in real samples was accomplished in both tap and drinking water samples. The reliable R.S.D. values and recoveries demonstrated that sensor **BK** had a valuable potential for being used as a dependable tool to monitor ClO^−^ in real water samples (Table 1).

### 3.2. In Vivo Imaging in Zebrafish

**Figure 8:**
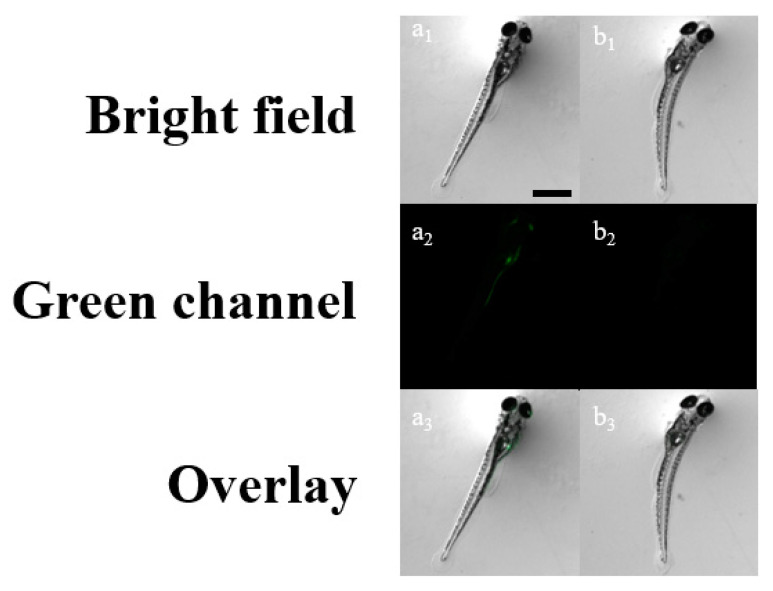
Fluorescence images of zebrafish treated with **BK** followed by addition of ClO^−^ (λ_ex_ = 502 nm and λ_em_ = 526 nm). (**a_1_**–**a_3_**): **BK** only; (**b_1_**–**b_3_**): **BK** with 5 × 10^−5^ M ClO^−^. [**BK**] = 2 × 10^−5^ M. Scale bar: 0.89 mm.

To test whether the probe **BK** is applicable under biological conditions, zebrafish were incubated with **BK** (20 μM) and sequentially treated with two different concentrations of ClO^−^ (0 and 50 μM) for imaging (Figure 8). Zebrafish incubated with **BK** exhibited a green fluorescence image, but the green fluorescence was eliminated in the presence of ClO^−^. Meanwhile, the cytotoxicity test of **BK** was examined by AO staining (Figure S7). The AO stained results showed that no apoptosis was observed in control and the presence of **BK** and 9-aminoacridine. Zebrafish experiments proved that **BK** was organism-permeable and can monitor ClO^−^ in living organisms. Importantly, **BK** is the first fluorescent turn-off sensor capable of sensing ClO^−^ in zebrafish (Table S1) [1,38,54,55,56,57,58,59]. 

## 4. Conclusions

We have synthesized an acridine-based chemosensor for monitoring ClO^−^ in a near-perfect aqueous media. Probe **BK** selectively detected ClO^−^ over other relevant species including ROS, and its intense blue fluorescence was notably quenched with the addition of ClO^−^. The detection limit of **BK** for ClO^−^ was analyzed to be 7.65 μM. **BK** was employed for quantitative measurement of ClO^−^ in real water samples and zebrafish. Significantly, **BK** is the first fluorescent turn-off sensor capable of sensing ClO^−^ in zebrafish to date. The promising outcomes indicate that **BK** can serve as a potential chemosensor for monitoring ClO^-^ in chemical, environmental and biological systems. We believe that these results will be merited for further development of ClO^−^ sensors.

## Figures and Tables

**Table 1:** Determination of ClO^−^.^a.^

Sample.	ClO^−^ Added (µM)	ClO^−^ Found (µM)	Recovery (%)	R.S.D. (n = 3) (%)
Drinking water	0.00	0.00		
	60.00 ^b^	62.88	104.80	0.32
Tap water	0.00	0.00		
	60.00 ^b^	62.16	103.60	0.41

^a^ Conditions: [**BK**] = 10 µM in bis-tris buffer. ^b^ 60.00 µM of ClO^−^ was artificially added.

## Supplementary Materials

The following are available online at https://www.mdpi.com/1424-8220/20/17/4764/s1, Table S1: Examples of fluorescent chemosensors for detecting ClO^−^ in zebrafish, Figure S1: Determination of the detection limit for ClO^−^ by **BK** (10 μM) based on the fluorescence emission at 455 nm; Figure S2: Negative-ion electrospray ionization mass spectrum of **BK** (10 μM) upon addition of NaClO (200 equiv); Figure S3: Molecular orbital diagrams and excitation energies of (a) BK and (b) ACR; Figure S4: (a) The theoretical excitation energies and the experimental UV-vis spectrum of BK. (b) The major electronic transition energies and molecular orbital contributions for BK (H = HOMO and L = LUMO); Figure S5: (a) The theoretical excitation energies and the experimental UV-vis spectrum of ACR. (b) The major electronic transition energies and molecular orbital contributions for ACR (H = HOMO and L = LUMO); Figure S6. (a) The theoretical excitation energies and the experimental UV-vis spectrum of ACR. (b) The major electronic transition energies and molecular orbital contributions for ACR (H = HOMO and L = LUMO); Figure S7. AO-stained zebrafish exposed to (a) 0 μM and (b) 20 μM of BK and (c) 20 μM of 9-aminoacridine.
