# Supplementary material for: An Acridine-Based Fluorescent Sensor for Monitoring ClO− in Water Samples and Zebrafish
Source: Sensors (Basel). 2020 Aug 23;20(17):4764. doi: 10.3390/s20174764 (PMC7506904; doi:10.3390/s20174764)
Supplement: Supplementary file 1 [file sensors-20-04764-s001.pdf]

sensors

MDPI

Letter

Supporting Information

An Acridine-Based Fluorescent Sensor for Monitoring ClO^- in Water Samples and Zebrafish

Su Chan Lee [1], Soyoung Park [1], Haeri So [1], Gyudong Lee [2], Ki-Tae Kim [2,*] and Cheal Kim [1,*]

[1] Department of Fine Chemistry, Seoul National University of Science and Technology, Seoul 136-741, Korea; rlthro456@naver.com (S.C.L.); pp113833@hanmail.net (S.P.); gofl0988@naver.com (H.S.)
[2] Department of Environmental Engineering, Seoul National University of Science and Technology, Seoul 136-741, Korea; rbehd8024@gmail.com
* Correspondence: ktkim@snut.ac.kr (K.-T.K.); chealkim@snut.ac.kr (C.K.); Tel.: +82-2-960-6683 (K.-T.K. and C.K.); Fax: +82-2-971-9139 (C.K.)

Table S1. Examples of fluorescent chemosensors for detecting ClO^- in zebrafish.

No.	Structure	Detection limit	Reaction media	Method of detection	Reference
1	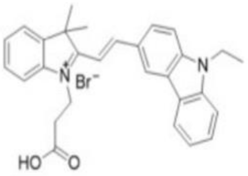	0.31 μM	DMSO/H_2O=5:1	colorimetric ratiometric	[54]
2	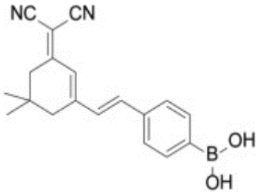	0.0157 μM	20% ethanol solution	ratiometric	[55]
3	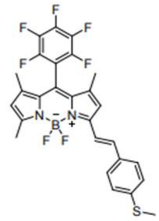	0.059 μM	DMF:PBS buffer=1:1	ratiometric	[56]
4	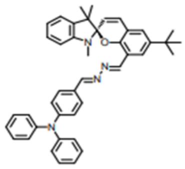	0.0642 μM	DMSO/H_2O=5:1	fluorescence turn-on colorimetric	[1]
5	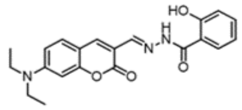	0.128 μM	EtOH/H_2O =1:19	fluorescence turn-on colorimetric	[38]
6	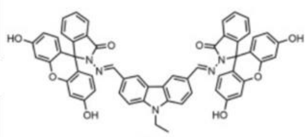	0.056 μM	PBS buffer:DMSO=99:1	fluorescence turn-on	[57]

7	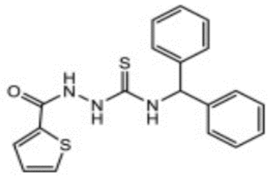	0.28 μM	PBS buffer (pH 7.4, 10 mM)	fluorescence turn-on	[58]
8	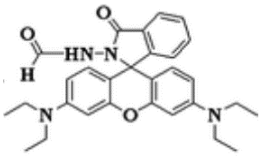	0.116 nM	PBS buffer (pH 7.4, 10 mM)	fluorescence turn-on	[59]
9	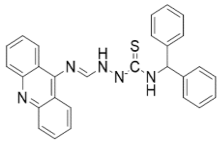	7.65 μM	Bis tris buffer	fluorescence turn-off	This work

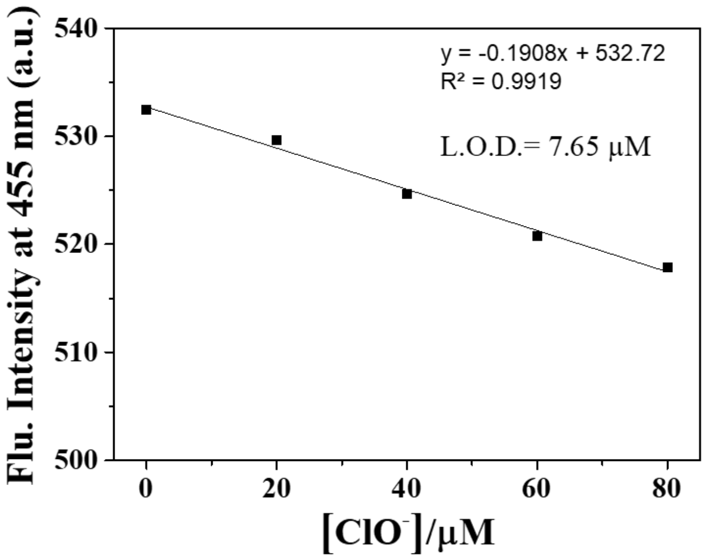

Figure S1. Determination of the detection limit for ClO^- by **BK** (10 μM) based on the fluorescence emission at 455 nm.

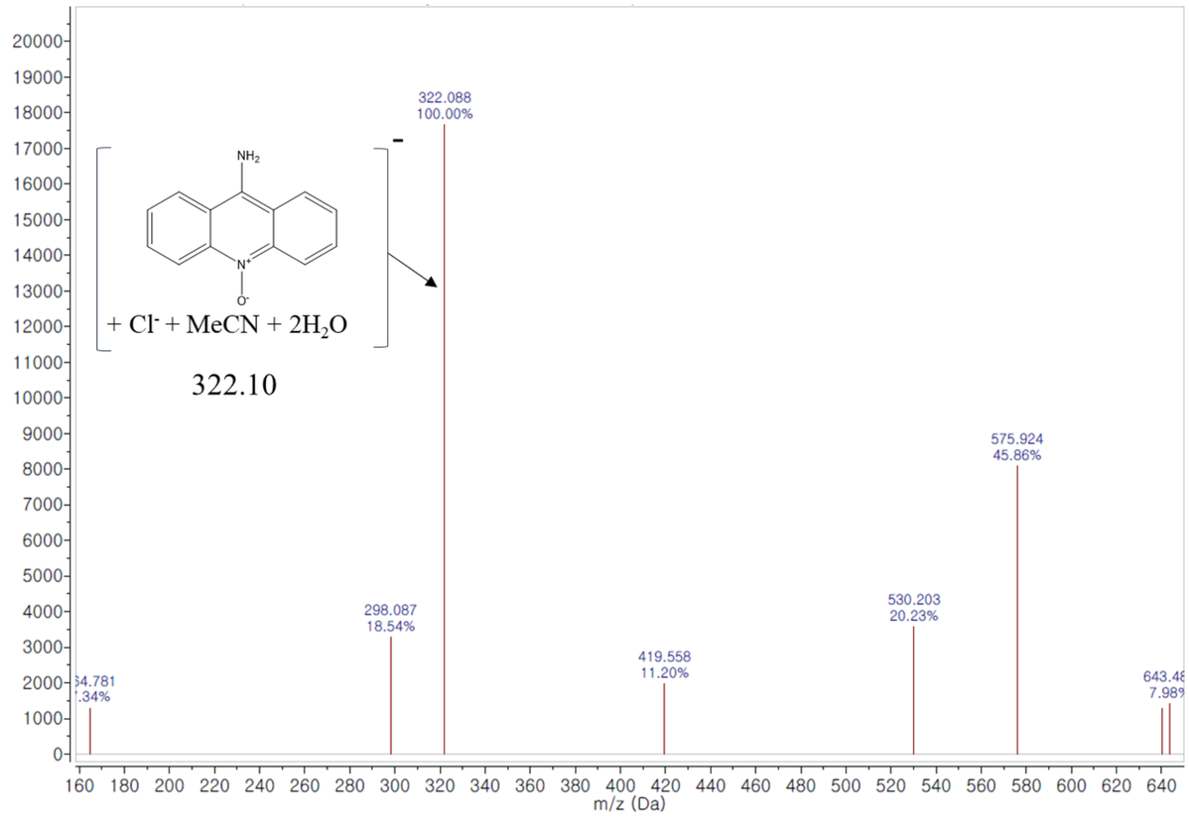

Figure S2. Negative-ion electrospray ionization mass spectrum of **BK** (10 μM) upon addition of NaClO (200 equiv).

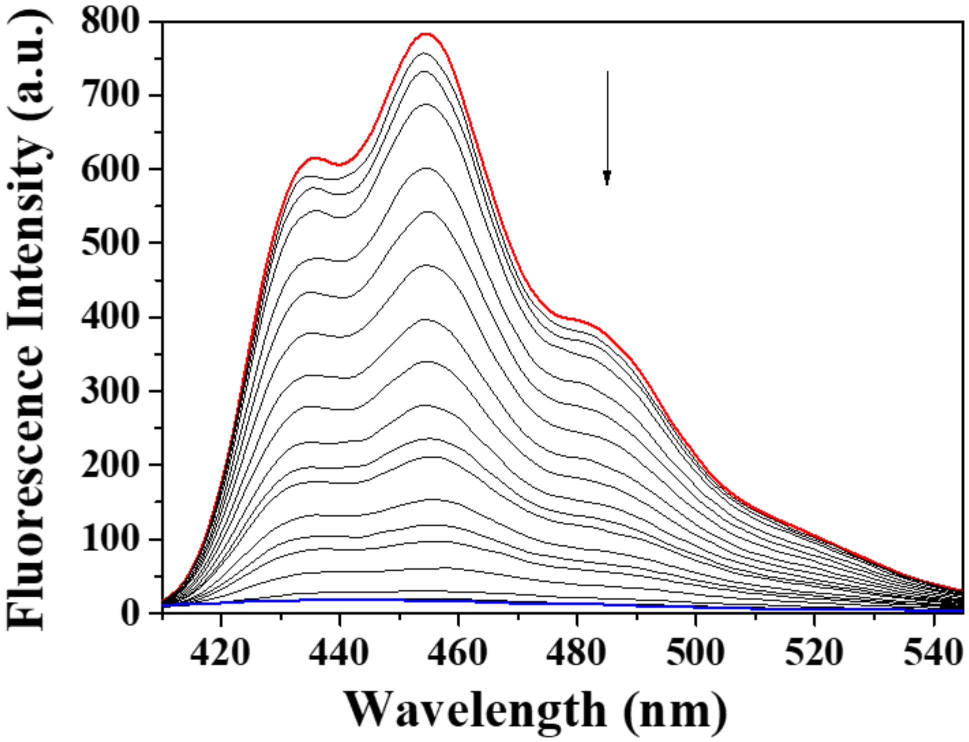

Figure S3. Fluorescent change of **ACR** with different concentrations of ClO^-.

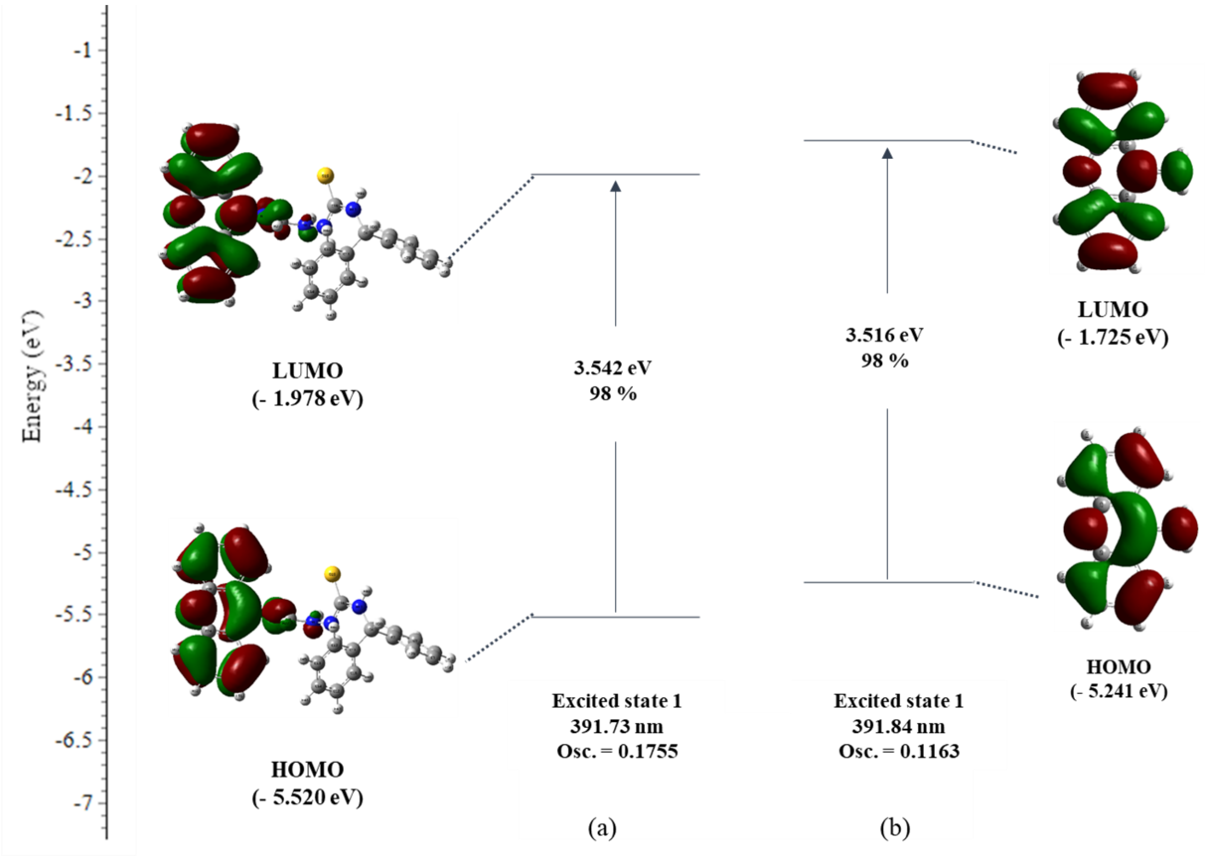

Figure S4. Molecular orbital diagrams and excitation energies of (**a**) **BK** and (**b**) **ACR**.

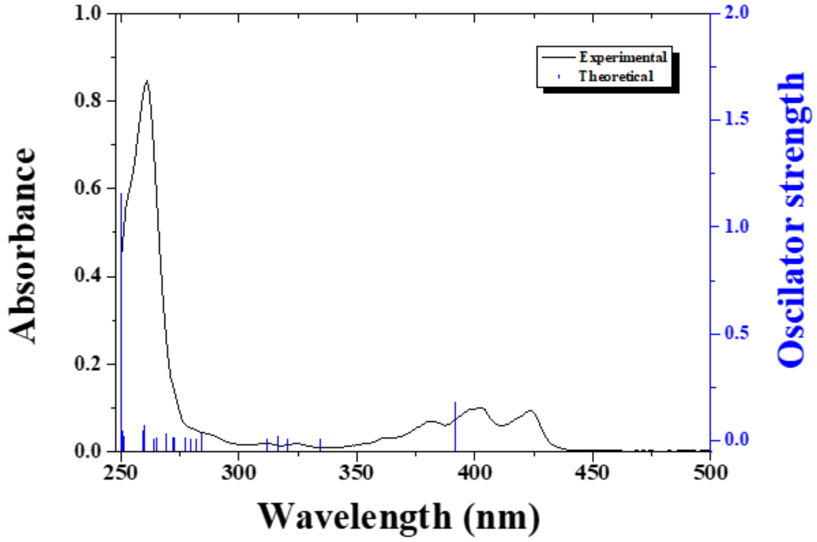

(**a**)

Excited State	Wavelength	Percent, %	Character	Oscillator strength
H → L	391.73 nm	98 %	π → π* transition	0.1755

(**b**)

Figure S5. (**a**) The theoretical excitation energies and the experimental UV-vis spectrum of **BK**. (**b**) The major electronic transition energies and molecular orbital contributions for **BK** (H = HOMO and L = LUMO).

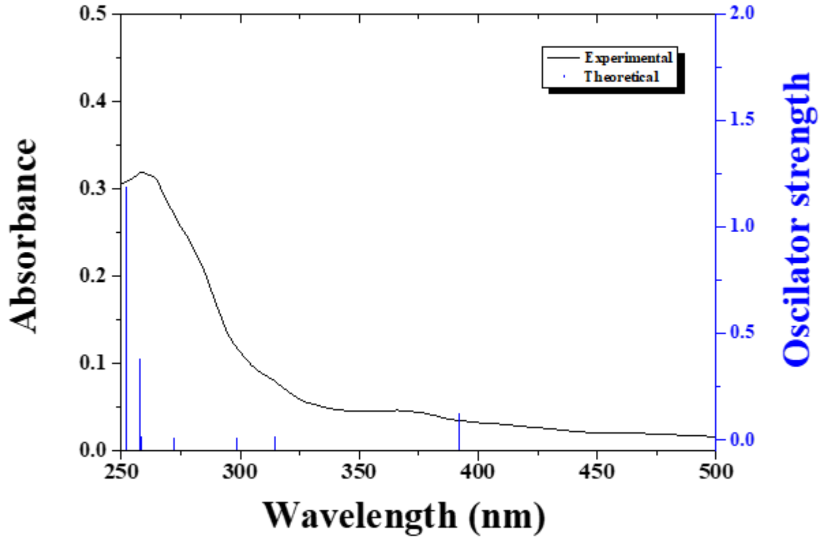

(**a**)

Excited State	Wavelength	Percent, %	Character	Oscillator strength
H → L	391.84 nm	98 %	$\pi \rightarrow \pi^*$ transition	0.1163

(**b**)

Figure S6. (**a**) The theoretical excitation energies and the experimental UV-vis spectrum of **ACR**. (**b**) The major electronic transition energies and molecular orbital contributions for **ACR** (H = HOMO and L = LUMO).

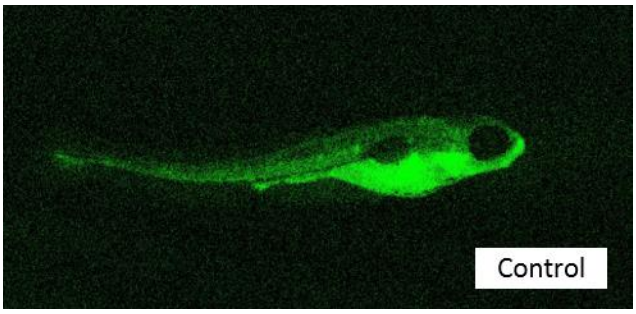

(**a**)

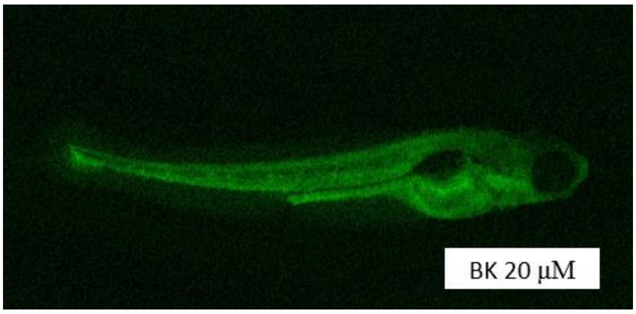

(**b**)

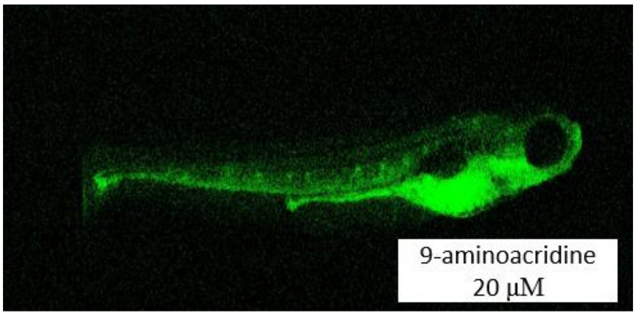

(**c**)

Figure S7. AO-stained zebrafish exposed to (**a**) 0 μM and (**b**) 20 μM of **BK** and (**c**) 20 μM of 9-aminoacridine.
